# Adapting an evidence-based mindfulness-based intervention for sheltered youth experiencing homelessness

**DOI:** 10.1186/s12906-023-04203-5

**Published:** 2023-10-17

**Authors:** Diane Santa Maria, Paula Cuccaro, Kimberly Bender, Erica Sibinga, Natalie Guerrero, Najiba Keshwani, Jennifer Jones, Micki Fine

**Affiliations:** 1 University of Texas Health Science Center at Houston Cizik School of Nursing, 6901 Bertner Ave, Houston, Texas 77030 United States; 2 Health Promotion and Behavioral Sciences, University of Texas Health Science Center at Houston School of Public Health, Houston, USA; 3 Graduate School of Social Work, University of Denver, Denver, USA; 4 Department of Pediatrics, Johns Hopkins University School of Medicine, Baltimore, USA; 5 Department of Pediatrics, Baylor College of Medicine, Houston, USA; 6 Center for Nursing Research, University of Texas Health Science Center at Houston Cizik School of Nursing, Houston, USA; 7 Certified Mindfulness-Based Stress Reduction Teacher by University of Massachusetts Medical Center Mindful Living, Worcester, USA

**Keywords:** Youth homelessness, Mindfulness based interventions, ADAPT-ITT, Intervention adaptation, Youth shelters

## Abstract

**Objectives:**

Youth experiencing homelessness (YEH) face challenges that impact their physical, mental, and social wellbeing, emotion regulation, and coping. Mindfulness reduces stress and improves resilience, emotion regulation, and executive functioning. Mindfulness-based interventions (MBI) teach the practice of mindfulness to foster present-moment attention without judgement and enhance self-observation and self-regulation, resulting in greater awareness of thoughts and emotions and improved interpersonal relationships. One such intervention, .*b*, has been shown to lower stress among youth. While a pilot study of .*b* among sheltered youth found the intervention to be feasible, the need for modifications was identified to improve its relevance, accessibility, and incorporate a trauma-informed approach.

**Methods:**

We used the ADAPT-ITT (Assessment, Decisions, Administration, Production, Topical experts, Integration, Training staff, and Testing) framework to adapt the .*b* mindfulness intervention to YEH living in an emergency shelter. Nine focus group discussions (n = 56), key informant interviews (n = 8), and beta testing with five youth working group sessions (n = 10) identified needed modifications.

**Results:**

Adaptations to the curriculum and delivery modality were made to approximate the average length of stay in the shelter; integrate trauma-informed care approaches; increase diversity of images by race, ethnicity, age, sexual orientation, and gender identity; and increase the relevance of the audio-visual components.

**Conclusions:**

Youth and the health and social services providers who care for youth generally liked the core concepts and presentation of the curriculum. Using the ADAPT-ITT framework, minor, yet important, changes were made to increase the relevance, acceptability, and feasibility of the intervention. Next steps are to conduct a randomized attention control pilot study to assess feasibility and acceptability.

## Introduction

On any given night in the U.S., up to 3.5 million youth under age 25 experience homelessness [1–4]. Youth experiencing homelessness (YEH) suffer worse physical and mental health than the general adolescent population and have a mortality rate 10 times higher than housed youth, with suicide and drug overdose being the leading causes of death [5, 6]. Often, YEH arrive on the streets with a history of adverse childhood experiences and are more likely to have grown up in households with parents or caregivers who had mental health disorders, substance use issues, psychiatric disorders, and criminal involvement [7, 8]. It is also important to note that systemic failures contribute to this lack of wellbeing among unhoused young people, including inequitable access to housing, healthcare, employment, and education [9]. Despite recognized disparities in outcomes for YEH compared to other youth, they remain an underserved population across health and social services.

Often in response to high levels of trauma and stress, YEH engage in high-risk behaviors including substance use and condomless sex [10–13]. The effect of stressful events and toxic stress on youth’s psychological development impacts the ways they learn to cope with stress [14, 15]. Managing discomfort and symptoms through adaptive coping mechanisms may ultimately reduce daily stress to momentary disruptions whereas other forms of coping, such as engaging in substance use and risky sexual behaviors, may exacerbate stress and place young people at additional risk for negative health outcomes [16–20]. Youth who utilize riskier coping strategies, and who experience high stress and/or low social support are at greater risk for depression, co-morbidities, substance use, and condomless sex [15, 21–24]. Moreover, the chronic stress of past trauma and current homelessness is exacerbated by prevailing mood disorders [25]. Specifically, YEH experience anxiety disorders nearly 2 times, major depressive disorder 2 to 3 times, and post-traumatic stress disorder at 3.5 to 13 times the rate of housed peers [26]. Improving stress management in YEH is therefore a key opportunity to reduce these unacceptably high rates of suicide, substance use, and comorbidities.

Increasing healthy life skills such as stress management, emotion regulation, and executive function may optimize the capacity of YEH to make health-promoting decisions and thus decrease risk behaviors and rates of suicide, substance use, and other comorbidities. To this end and because of the high levels of past trauma and mental health needs, interventions targeting YEH must use a trauma-informed model that addresses the state of vulnerability, high stress, unstable housing, trauma, and compromised executive functioning that many youth experience to increase their resilience (i.e., capacity to cope successfully) and reduce life-threatening behaviors [27, 28].

Mindfulness-based interventions (MBI) aim to enhance the innate capacity of mindfulness, fostering present-moment attention without judgement and increasing self-observation and self-regulation, resulting in greater awareness of thoughts and emotions, reduced reflexive reactivity, and improved impulse control and interpersonal relationships [29–31]. Mindfulness reduces stress while improving resilience, self-regulated behaviors, and executive function [32–35]. Mindfulness approaches build skills to increase non-judgmental attention and emotional reappraisal and improve present-focused states of experiencing cognitions and emotions that lead to less reflexive reactivity, reduced impulsivity, greater contemplation of behaviors, and improved interpersonal dynamics and relationships [36, 37]. Evidence demonstrates the efficacy of MBIs across various populations and outcomes, including improved affect and executive functioning and, among adolescents and emerging adults living with HIV, mindfulness instruction improved HIV medication adherence through greater self-awareness and self-acceptance [38–44]. Several studies across youth populations have found that mindfulness practice reduces stress and improves self-regulated behaviors, executive function, and resilience toward stress [35, 38–41, 45–49].

Evidence of the effectiveness of mindfulness approaches in adolescents is mounting, demonstrating increased mindful attention and awareness and decreased reactivity in school-aged youth; enhanced self-regulation and coping among youth; improved mental health, emotional control, and reduced post-trauma stress symptoms in urban youth; and reduced stress and anxiety in adolescent psychiatric patients [48, 50, 51]. Reviews of youth-based MBIs and meditation practices suggest that these interventions may also lead to improvements in depression and anxiety [52, 53].

**Fig. 1:**
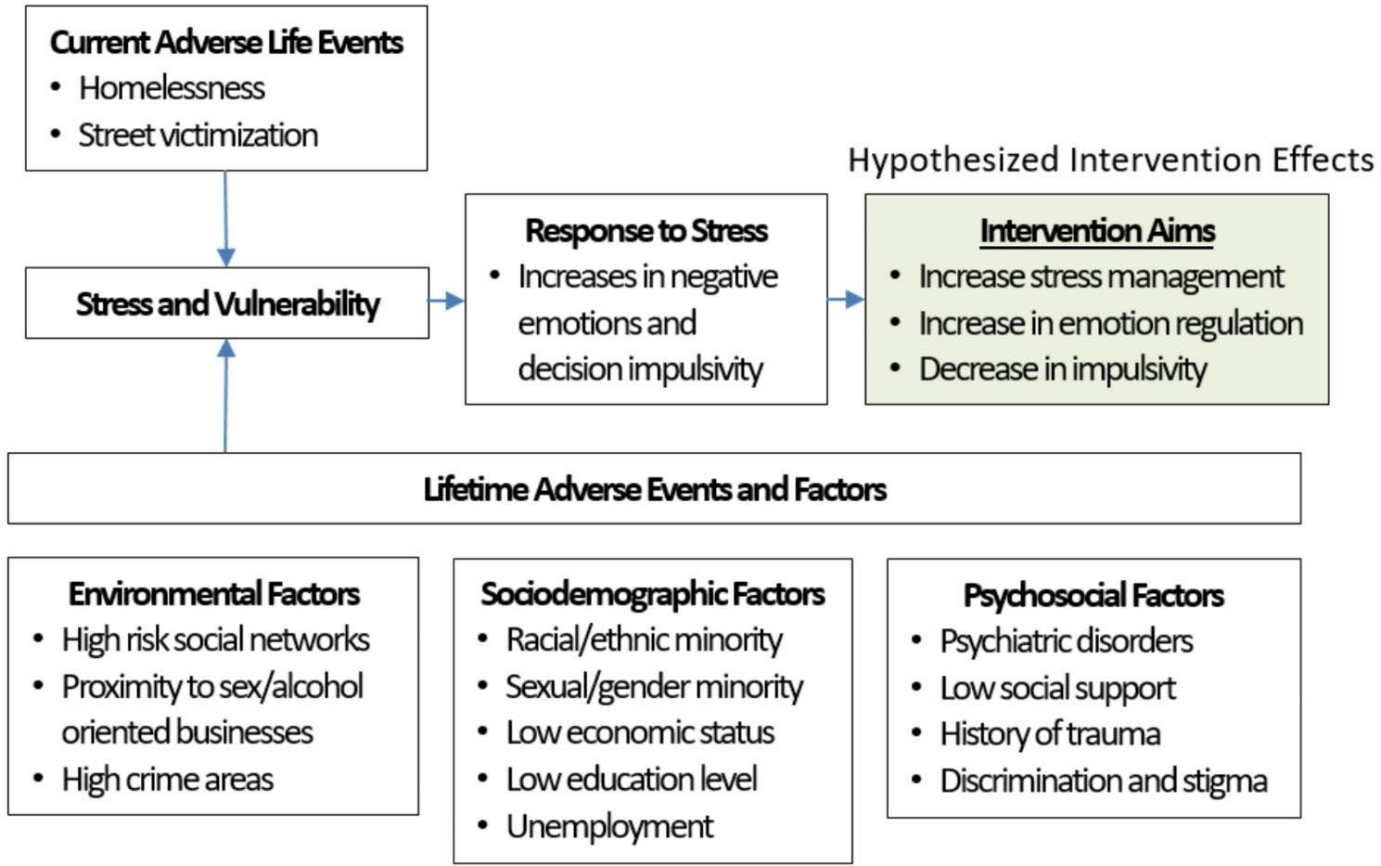
Theoretical Framework Guiding Intervention Adaptations

While mindfulness has been used with other high-risk, high-trauma youth populations, there is a dearth of published research on mindfulness strategies among sheltered YEH. Therefore, we developed a theoretical framework informed by the Risk Amplification Model (RAM) and the minority stress model (MSTM) to guide this research [11, 54]. This framework accounts for the trauma and stress resulting from minority status, socio-demographic, environmental, and psychosocial factors, which serve as negative contacts and socializing agents that amplify risk (Fig. 1) [55]. Subsequently, that trauma and stress intersect with the vulnerabilities of homelessness, influencing the development of negative emotions, reactive stress responses, and impulsive decision making that can lead to risky behaviors and are prevalent among YEH. Therefore, this framework takes into consideration the environmental, sociodemographic, and psychosocial factors contributing to the high level of vulnerability and stress experienced by YEH.

### ***.b*** Intervention

A mindfulness based intervention, .*b* (pronounced dot be), was chosen due to its promising results and relatability to youth. .*b* originated from Mindfulness in Schools Project (MiSP) in the U.K. and is often delivered in groups at schools over nine weekly 45-minute sessions that consist of lessons addressing emotional awareness, sustained attention, attention regulation, and emotion regulation (Huppert & Johnson, 2010). .*b* is based on the tenets of the evidence-based mindfulness based stress reduction and mindfulness based cognitive therapy [56, 57]. A randomized trial of .*b* found lower stress levels (p = 0.05) in youth after receiving the curriculum and high levels of acceptability among the sample of 522 school-based youth aged 12–16 years with mindfulness practice being associated with less stress (p = 0.03) [58].

Guided by the theoretical framework, we present the process used to adapt the .*b* curriculum to increase the acceptability and feasibility of testing this intervention among a population of sheltered YEH. This intervention will be designed to be delivered in partnership with housing and shelter providers [59–61].

## Method

The ADAPT-ITT (Assessment, Decision, Adaptation, Production, Topical experts, Integration, Training staff, and Testing) framework is a process by which interventions may be adapted to new populations and settings (Wingood & DiClemente, 2008) and was used to guide this study [62]. Although initially used to adapt HIV interventions, the framework has been widely used to adapt other interventions, such as a sexual violence primary prevention program [63]. We describe the use of the framework to adapt the .*b* curriculum to a population of youth temporarily residing in a shelter. This study was reviewed and approved by the University of Texas Health Science Center Committee for the Protection of Human Subjects. All participants signed informed consents.

### Phase I: Assessment

The assessment phase was used to understand the priorities and needs of the community and population. This phase included reviewing the results of our prior mixed methods feasibility study. In the pilot study, we tested the feasibility and acceptability of using a slightly modified version of the .*b* curriculum among 39 sheltered YEH aged 18–21 years [64]. Though the program is usually delivered in a school setting over multiple weeks, in the shelter environment, in the pilot study, we had to make modifications to approximate the average time YEH spend in a shelter. Therefore, we delivered the lessons in five 90-minute long sessions over 2.5 weeks. Though the results of the pilot intervention showed feasibility, findings also suggested that more substantial modifications were needed to improve its relevance and accessibility of the intervention for YEH and to ensure a trauma-informed approach [64]. In this paper, we present the adaptation process of the .*b* curriculum to .*b4me* – a version tailored to YEH.

As the previous pilot study demonstrated initial feasibility with the target population, the next step entailed modifying the curriculum to improve relevance along with the delivery of the lessons. First, the adaptation team received in-depth, formal, 3-day facilitator training of the .*b* intervention through the MiSP .b training program. Next, the research coordinator and the research assistant facilitated nine one-hour focus group discussions with YEH at a local shelter between March and April, 2021. A total of 56 participants were recruited through distributed flyers and onsite study recruitment events at shelters and drop-in centers. Focus group discussions were held in the conference room or library of the youth homeless shelter to improve ease of participation. After participants completed the informed consent process, they took a demographic survey and then attended a focus group session. Participants received a $20 gift card for their participation in the focus groups.

### Phase II: Decisions

In the second phase of the ADAPT-ITT process, we reviewed the extant literature to inform what additional adaptations to the current intervention program were needed. We compiled studies focused on curriculum content and delivery areas identified in the original .*b* curriculum, and through the pilot study, as potentially needing modification through the adaptation process. During the adaptation process, we prioritized these areas of modification to increase the relevance and accessibility of the intervention to sheltered YEH.

### Phase III: Adaptation

We aimed to improve the way the intervention was delivered by beta-testing iterations of the adaptations with a Youth Working Group (YWG) using a theater testing approach [62]. YEH (n = 10) were included in the YWG and were recruited through a convenience sample of youth residing in the shelter and received a $20 gift card for participating in each session. During these sessions, we tested modified session content, delivery methods, and strategies. YWG participants were briefed about the goals and objectives of the lesson at the beginning of each session. Following a presentation of proposed session activities, participants provided input about the materials, whether the delivery methods and strategies used to present the materials were effective. Additionally, cognitive interviews with YEH were completed to assess the understandability of the wording of the proposed outcome measures. A total of five YWG sessions were held at the youth shelter, each lasting approximately two hours in length. Youth in the YWG were also asked about various commercially available apps they may have used prior or would be interested in using as part of the intervention to enhance continued practice of the mindfulness strategies introduced in the curriculum.

### Phase IV: Production

In the production phase, materials needed for the intervention program were created, including revisions to the intervention facilitator scripts, new illustrative video clips, and media to replace original materials that were not relevant. These changes were integrated into the final presentation modules. The study team also began production of the revised facilitator manual, which was also informed by the administration/adaptation phase.

### Phase V: Topical Experts

This phase provided the opportunity for input from topical experts. Experts were engaged from Phase III through finalization of the adapted curriculum in phase VI through key informant interviews. These experts were identified based on their work with YEH (i.e., social worker, resident advisor), expertise in MBIs (i.e., MBSR teacher), and understanding of trauma informed approaches (i.e., mental health providers). Key informants were also interviewed to review suggested adaptations and solicit their feedback. Finally, an advisor from the curriculum developer, MiSP, was involved throughout the adaptation process. Interviews were conducted with nine key informants to gather feedback on the proposed adaptations and elicit suggestions of ways to improve the feasibility of conducting the subsequent randomized trial, and consider commercially available mindfulness apps that youth may find helpful. Additionally, an interdisciplinary Expert Advisory Panel (EAP) was created with the goal of providing feedback on the proposed changes and advising the team on intervention development, implementation, evaluation, and study design and protocol development.

### Phase VI: Integration

This phase entailed integration of feedback received from the youth in the focus groups, YWGs, and the key informant interviews into the adapted intervention and finalization of the facilitator’s manual and curriculum materials.

### Phase VII: Training

The training phase involved the thorough training of three .*b* intervention facilitators who implemented the adapted intervention during the pilot study. Facilitators, who were certified MBSR instructors and had a formal mindfulness practice, first participated in a three-day facilitator training on the original .*b* curriculum. Once all adaptations were completed, the facilitators were trained on the delivery of the adapted curriculum, .*b4me*.

### Phase VIII: Testing

In the testing phase (currently in process), we are conducting a pilot randomized attention controlled feasibility trial of the .*b4me* intervention among sheltered youth. The manualized control condition *Healthy Topics* is a previously tested curriculum about healthy habits for adolescents used in the authors’ prior work [38, 39]. The session number and length equal that of the .*b4me* curriculum.

### Analysis

A loosely structured interview guide was used for the focus groups, key informant interviews, and YWG sessions, which were recorded and professionally transcribed. ATLAS.ti software version 8.6 was used to organize the themes and codes that emerged from these qualitative methods. The team used thematic content analysis to develop codes, identify patterns in the data, and generate themes to label those key data patterns [65, 66]. Using an iterative process, three team members coded all transcripts and then organized the data into text groupings and aligning those groupings into themes. To represent the themes, exemplar quotes were identified [67]. We used full team and peer debriefing with the YWG to enhance the trustworthiness and credibility of the analytic process and interpretation of the findings [68]. All authors provided feedback on data interpretation throughout the process.

#### Focus Group Discussions

Audio recordings for nine focus group discussions were transcribed verbatim by an independent transcription service. Three individuals served as coders: a graduate research assistant, an undergraduate honor’s research student, and a research assistant experienced in qualitative research data collection and analysis. To guide the coding process, the team looked for data on what youth reported as adaptations that would increase the relevance of the .*b* curriculum materials for youth living in a homeless shelter. The main data coders first agreed on a priori codes based on the theoretical framework, then thematic content analysis was used to develop codes derived from the transcripts. The team first group coded the transcript with the most content and then each independently coded all nine focus group transcripts. The team then met to review each transcript and individual codes to reach a consensus on codes and themes and develop a codebook based on the theoretical framework and data that emerged from the transcripts.

## Results

Table 1 outlines the application of ADAPT-ITT to this study including a summary of methods and results for each phase.

**Table 1:** Applying the ADAPT-ITT Framework to a Mindfulness Intervention for Youth Experiencing Homelessness

Date	ADAPT-ITT phase	Phase objective	Methodology	Results
Fall 2015-Winter 2016 and Spring 2021	I. Assessment	Understand the priorities and needs of YEH living in a shelter	Review pilot of original .b and conduct focus groups with 56 YEH	Original .b was found to be acceptable; modifications are needed to enhance cultural relevance and accommodate average length of stay at shelter
Fall 2015-Winter 2016 and Winter 2020-Spring 2021	II. Decisions	Assess preexisting curricula (.b) and determine suitability for adaptation	Review previous pilot study results, and feedback from focus group	Determined that adaptations to .b would enhance relevance of content for YEH
Fall-Winter 2021	III. Administration/ Adaptation	Administer proposed adaptations of .b to YEH	Beta-test with Youth Working group. Total participants = 10	YWG confirmed acceptability of the vast majority of adaptations, and made suggestions for slight modifications
Fall-Winter 2021	IV. Production	Produce adapted version of .b curriculum to test with YEH	Compile adaptations in preparation for integration into facilitator’s manual and PPT slides	Draft of *.b4me* facilitator’s manual and materials
Fall-Winter 2021	V. Topical Experts	Review adaptations with experts in youth homelessness, mindfulness, and/or trauma-informed care	Convene EAP, conduct key informant interviews, and consult MiSP representatives to review adaptations	Identified themes related to barriers, facilitators, and other suggestions.
Spring 2022	VI: Integration	Integrate feedback from experts with proposed adaptations and finalize adapted version of .b curriculum	Compile feedback from EAP, key informants, and finalize draft of manual and materials for final approval from MISP	Final version of the .*b4me* facilitator’s Manual and materials
Spring 2022	VII: Training	Train facilitators to implement the adapted program with YEH	Provide an overview of adapted .b curriculum to facilitators trained in original .b program	2 trained interventionists
Spring 2022—Fall 2022	VIII: Testing	Test the adapted program with YEH	Conduct a pilot RCT (n = 60) with YEH	Results pending completion of RCT.

NOTE: YEH = youth experiencing homelessness; YWG = youth working group; PPT = power point; EAP = expert advisory panel; MiSP = mindfulness in schools program; RCT = randomized controlled trial

### Phase I: Assessment

The focus groups conducted in the assessment phase allowed us to get insight on what residents at the shelter saw as culturally relevant. A total of sixty-two codes were used throughout the transcripts. Codes were organized into two categories: general mindfulness and suggested adaptations. The themes within the mindfulness category were: mindfulness, meditation, and spirituality. Overall feedback related to suggested adaptations were in response to preferring newer materials to outdated material, unfamiliar cultural references, and sensitive topics. Ten themes emerged that fall within the adapted curriculum topics: training the mind, living in the present, mindfulness in sports, taming the animal mind, recognizing worry, being in the zone, stepping back, taking in the good, recognizing and dealing with anxiety, and connection between thoughts, feelings, body sensation, and action. The team met weekly to review the themes in transcripts. The full study team then used those findings to guide decisions regarding which adaptations would be presented to the YWG participants.

### Phase II: Decisions

Adaptations to the .*b* original curriculum that were presented to the YWG included modifications aimed at improving cultural relevance and increasing the sense of safety while implementing the modified activities. Specifically, participants agreed unanimously to replace cultural references to United Kingdom (where .*b* was developed), older videos depicting core concepts with more updated video clips with relevant artists or athletes, and to create original content (described below) that was more relatable to the life experiences of YEH and staying in a shelter.

### Phase III: Adaptation

A total of 10 gender, racially, and ethnically diverse YEH recruited from the shelter participated in the YWG. Findings from each round of iterative testing of the proposed curriculum changes further informed modifications to the survey instruments, session materials, and delivery methods and strategies that were further tested in subsequent rounds of theater testing with the YWG. YWG sessions were concluded once participants reported a high level of acceptability and relevance with the content adaptations. Using cognitive interviewing strategies, youth also offered feedback on the understandability of the proposed outcome measures that the team then used to determine the preferred scales and items to be used in the trial baseline and follow-up surveys. To address the need to replace some video content to improve the relevance of a gratitude activity, the YWG recorded their thoughts on what mindfulness and gratitude meant to them and offered genuine advice to other youth experiencing homelessness. This replaced a video discussing Thanksgiving that referenced healthy family connections. The youth felt that Thanksgiving was not always joyful for them and they wanted to describe other ways of feeling gratitude even when family and life situations are undesirable and challenging. None of the adaptations impacted the core materials from the original .*b* curriculum.

### Phase IV: Production

The .*b4me* facilitator’s manual and accompanying audio-visual materials were developed in this phase in collaboration with MiSP. This led to a .*b4me* facilitator’s manual and changes to the presentation materials associated with each of the sessions. An animation expert developed the new gratitude video content using the words of the youth derived during the YWG sessions in an animation video.

### Phase V: Key Informant Interviews and Expert Panel

Themes and examples from the expert informants’ interviews are displayed in Table 2. Informants included a deputy director and counselors in juvenile probation, adolescent medicine physicians, a psychologist specializing in mindfulness-based treatment, homeless prevention and service providers, and an executive director of a local homeless shelter. Resulting information included barriers and facilitators to program implementation, suggestions for increasing youth engagement, recommendations for creating a safe and welcoming space, recommendations on which mindfulness apps are youth friendly, insight on YEH’s prior exposure to mindfulness concepts, potential challenges with new strategies or concepts, and ways to improve accessibility of the curriculum. Additionally, an Expert Advisory Panel that included five homeless youth service providers, recruited from the Homeless Youth Network, met to review and discuss the proposed curriculum adaptations.

**Table 2:** Themes and Examples from Topical Experts

Theme	Example
Barriers to implementation	“Attendance would be the biggest one…and I don’t think it’s actually possible to say, okay, we’re going to avoid triggering. Like it’s going to happen, but it’s more how do we take care of it around them?” “anger management is a big deal with these kids”
Facilitators to implementation	“sometimes evenings are best” “physical movement, that playful engagement, that there’s an actual activity where…the brain is literally disconnecting from the body”
Suggestions for engagement	“The relevance for their own lives is the most important thing” “it’s getting them to not think this is something weird with a new dialogue because it’s a different language”
Recommendations for creating a more trauma informed approach (i.e. safe and welcoming)	“…always give kids a choice…use very invitational language” “…having the right sensory stimulation, having the materials that we need to set the environment and the scene for the kids to be able to relax” “…think about those…basic needs, water, nutrition, are they met? And thoughtful incentives”
Recommendations on mindfulness apps	“…it seems intimidating sometimes with the time. But if there are more options and maybe different categories”
Insight on YEH exposure to mindfulness	“Maybe they haven’t practiced it before, maybe they haven’t tried it, but they’ve heard about it” “The most relatable thing for them is religion. You know, prayer”
Anticipated challenges with new strategies or concepts	“I think there are certain meditation that can be quite difficult and that’s not to say that they shouldn’t be done, in my opinion, but they can be challenging because…when we sit with awareness, we notice what arises, and often what arises is discomfort. And so, I think it takes a lot of skill to be able to offer that in a trauma-informed way.” “…trying to not let external factors and external changes really affect you. You can only change yourself. So, I think it’s a concept that people grasp, but to put that into practice is really hard.”
Improving accessibility of the curriculum	“…emphasizing informal meditation is super important…much more accessible to them” “I think that’s important to say we’re not trying to fit you into a mold here. You know this is about what you want to do and how you want it to feel”
Overall suggestions	“…with a highly traumatized population, I think more somatic-based practices are more useful—more movement, less illness” “…having tangibles, having activities” “The key is not so much the tool, but the person who’s facilitating that conversation that person has to be very well trained and really clear and not easily triggered, which is really difficult”

### Phase VI: Integration

A total of 12 adaptations were made to the original .*b* curriculum and typical delivery modality. These adaptations included changing the delivery timeframe to approximate the average length of stay in the shelter which was about 21 days. Additional adaptations were the integration of trauma-informed care approaches to activities, increasing the diversity in race, ethnicity, age, sexual orientation, and gender identity among people and images in the audio-visual materials, and increasing the relevance of the audio-visual components by replacing with more contemporary examples.

To approximate the average length of stay in the shelter, the lessons were condensed from ten 40-60-minute lessons to five 60-90-minute sessions to be delivered over a three-week period. To further integrate trauma-informed approaches, the team worked closely with highly experienced interventionists on ways to cultivate a safe environment. Two main adaptations were made to content. One activity aiming to induce a stressful situation (use of a shock ball) was omitted due to the risk of retraumatizing participants. This activity was replaced with a youth suggested activity (tell your story to the group) that is anxiety provoking without being too intensely triggering for YEH with histories of trauma and abuse. As well, a new video was added normalizing that we sometimes experience dark thoughts but that they can come and go without acting on them. Six key replacements were made to images and audio-visual components to better represent the diversity and heterogeneity of YEH populations. Popular musicians, athletes, and movies suggested by the youth replaced references to films or figures that youth considered outdated or that more culturally aligned with youth from the UK versus the US. A video used in the original curriculum that described experiences and feelings of gratitude was replaced by an animated video created by youth describing what gratitude is like for them by using the words of the youth from the YWG. The audio for the video was recorded using voices other than the youths’ voices to protect their identities. These adaptations were integrated into the final version of the .*b4me* facilitator’s manual and presentation materials, which were approved and finalized by the creators of .*b*.

### Phase VII: Training

Three facilitators trained in the original .*b* curriculum and the adapted curriculum .*b4me* served as interventionists for the.*b4me* pilot trial. In addition to being trained in the original .*b* curriculum, the three interventionists were certified Mindfulness-Based Stress Reduction teachers, maintained a daily mindfulness practice, and had experience teaching mindfulness to adolescents and young adults. Additional training for .*b4me* included a comprehensive overview of the curriculum adaptations, study procedures for implementation, and frequent discussions with the study research team.

## Discussion

Despite the growing literature supporting the benefits of mindfulness for youth, to our knowledge, there is no other mindfulness intervention that has been specifically adapted in partnership with experts and youth to be delivered to youth who are temporarily seeking emergency shelter due to homelessness and housing instability. In this paper, we discuss the process and results of adaptations made to an evidence-based mindfulness intervention to increase the relevance and acceptability of intervention delivery to sheltered youth experiencing homelessness. Using the ADAPT-ITT model, we systematically modified aspects of the curriculum, in partnership with youth, experts in the field, and the curriculum developers while preserving the core content of the original, evidence-based program. In general, youth, shelter staff, and health and social services providers who care for youth experiencing homelessness approved of the core concepts and adapted materials and presentation of the curriculum, .*b4me*.

Minor, yet important, changes were made to increase the relevance, acceptability, and feasibility of the intervention. Utilizing both focus groups and the YWG sessions to elicit youth feedback was critical to enhancing the relevance and relatability of the content. Evoking suggestions from experts in the field on how to better incorporate trauma informed approaches into the content and delivery was a strength. The topical experts who participated in the adaptation process provided key considerations to the interventionists and study team on assuring a trauma-informed approach, increased the confidence and competence of the team to address challenges that may emerge, and helped create a safe and welcoming space for youth. Despite the challenges faced with the pandemic, a shelter relocation, and overall challenges of conducting research with this hard-to-reach population, we were able to gather critical feedback to inform the adaptation of .b4me.

This paper contributes to an ongoing conversation about intervention and implementation research. On one hand, several studies demonstrate that implementing interventions with fidelity to their core components and in adherence with their manuals (i.e., providing interventions as they were originally intended and tested) is essential to achieving intervention outcomes [69–71]. On the other hand, research also demonstrates that adaptation is often needed to make interventions more ecologically relevant and valid and to increase participant comprehension, salience, and motivation to engage [72]. However, some scholars argue that adaptations to established interventions are sometimes prematurely made based less on needed modifications but rather on practitioner’s comfort and familiarity with the intervention [73].

The ADAPT-ITT model offers a solution to this tension in that it preserves fidelity to core components of the model while systematically gathering data on necessary adaptations from intended recipients and other content experts [62]. In this case, using the ADAPT-ITT model resulted in an iteration of the .*b* mindfulness intervention tailored to the unique context, psychosocial needs, cultural norms, and the daily challenges of young people living in emergency shelter. Considering unhoused young people are often disaffiliated from most institutions and programs due to distrust of services that are experienced as inflexible and unresponsive, such systematic adaptation is necessary and warranted to adequately support sheltered young people in coping with the significant adversities they face daily [74–76]. Next, to accomplish Phase VIII: Testing, we are conducting a randomized attention-controlled pilot trial. This feasibility and acceptability trial will allow us to determine whether this adapted intervention can be feasibly delivered within the context and constrains of the shelter setting with youth who are currently receiving temporary shelter.

## Conclusion

The ADAPT-ITT model was a useful and appropriate framework to use in the adaptation of a mindfulness intervention for a new population and setting. Next, a randomized attention controlled trial will be conducted to assess the feasibility and acceptability of the new .*b4me* intervention.

## Data Availability

The datasets used and analyzed during the current study are available from the corresponding author on reasonable request.
